# A systematic review and meta-analysis on the effectiveness of xenograft to prevent periodontal defects after mandibular third molar extraction

**DOI:** 10.4317/medoral.24260

**Published:** 2020-10-09

**Authors:** Jorge Toledano-Serrabona, Víctor Ruiz-Romero, Octavi Camps-Font, Cosme Gay-Escoda, Mª Ángeles Sánchez-Garcés

**Affiliations:** 1 DDS. Fellow of the Master’s degree programme in Oral Surgery and Implantology, Faculty of Medicine and Health Sciences, Dental School, University of Barcelona. Researcher at IDIBELL (Bellvitge Biomedical Research Institute), Barcelona, Spain; 2 Dental student. Faculty of Medicine and Health Sciences, Dental School, University of Barcelona, Spain; 3 DDS, MS. Associate Professor of Oral Surgery. Master’s degree programme in Oral Surgery and Implantology, Faculty of Medicine and Health Sciences, Dental School, University of Barcelona. Researcher at IDIBELL (Bellvitge Biomedical Research Institute), Barcelona, Spain; 4 MD, DDS, MS, PhD, EBOS, OMFS. Chairman and Professor of Oral and Maxillofacial Surgery, Faculty of Medicine and Health Sciences, Dental School, University of Barcelona. Director of the Master’s degree programme in Oral Surgery and Implantology (EFHRE International University/FUCSO). Coordinator/Researcher at IDIBELL (Bellvitge Biomedical Research Institute). Head of the Oral Surgery, Implantology and Maxillofacial Surgery Department at the Teknon Medical Centre, Barcelona, Spain; 5 MD, DDS, MS, PhD, EBOS. Lecturer in Oral Surgery. Master’s degree programme in Oral Surgery and Implantology, Faculty of Medicine and Health Sciences, Dental School, University of Barcelona. Researcher at IDIBELL (Bellvitge Biomedical Research Institute), Barcelona, Spain

## Abstract

**Background:**

To evaluate the use of guided bone regeneration with xenograft to prevent periodontal defect in the distal aspect of the second molar after the surgical removal of the mandibular third molar.

**Material and Methods:**

Three electronic databases (Pubmed, Cochrane Library and Scopus) were searched in April 2020. Randomized clinical trials in non-smokers and healthy patients, with at least six months follow-up, comparing periodontal probing depth, clinical attachment level, alveolar bone level and adverse events were selected by two independent investigators. The risk of bias assessment of the selected studies was evaluated by means of the Cochrane Collaboration’s Tool. Finally, a meta-analysis of the outcomes of interest was performed.

**Results:**

Despite 795 articles were found in the initial search, only three randomized controlled clinical trials were included. Pooled results favoured the use of the xenograft plus collagen membrane over the spontaneous healing in terms of periodontal probing depth gain (MD=2.36; 95% CI 0.69 to 4.03; *P*=0.005) and clinical attachment level gain (MD=2.52; 95% CI 0.96 to 4.09; *P*=0.002). No other statistically significant differences were found.

**Conclusions:**

Within the limitations of the present review, the xenograft plus collagen membrane exhibited better periodontal results than spontaneous healing without increasing postoperative complications. However, future well-designed studies with larger samples are required to confirm our results.

** Key words:**Third molar, tooth extraction, bone regeneration, xenograft.

## Introduction

Extraction of mandibular third molar (M3M) is a very widespread practice in dentistry. Although its indication is clear when provoking symptoms or disease (e.g. infection, non-restorable caries, periodontal disease, root resorption), currently the prophylactic extraction remains a controversial issue (1). Indeed, the decision-making for removal of wisdom teeth has been discussed in the literature and some countries such as Finland, France, The United Kingdom or Spain, have made their own clinical practice guidelines, exhibiting discrepancies between them, especially about the prophylactic extraction of the M3M (2-4).

Periodontal disease on the mandibular second molar (M2M) is one of the primary reasons for the treatment of M3M (5). There are predisposing factors associated to the appearance of bony periodontal defects in the distal aspect of the M2M after the surgical removal of the M3M such as patient’s age (older than 25 years), position of the wisdom tooth or pre-existing periodontal defect. Knutsson *et al*. (6) described that mesioangular or horizontal M3M with a large contact with M2M had a greater risk of periodontal postoperative complications. Additionally, other studies have shown that the surgery itself can also cause a residual intrabony defect behind the M2M (7-9).

To prevent periodontal defects after the M3M extraction, various treatment modalities have been suggested, including different flap designs, soft-tissue suturing, and different bone and tissue regeneration techniques. In the context of periodontal regeneration therapy, bone substitutes such as autologous bone, allografts, xenografts or alloplastic grafts and occlusive membranes have been broadly studied (10,11). Each material is associated with some advantages and disadvantages, so their selection should depend on the clinical scenario, as well as, the preferences of the clinician and the patient (12).

Among these bone substitutes, the xenograft has been widely used in the field of bone reconstruction since it is a safe and a well-documented osteoconductive material with a low resorption rate. Due to its chemical composition and its trabecular structure, the xenograft has proven to be a good scaffold for cell growth, and thus, for bone regeneration (13,14).

Despite previous systematic reviews have been published on this topic, none of them compared solely the xenograft to the spontaneous healing. Thus, the aim of the present systematic review and meta-analysis was to gather the published randomized clinical trials to determine whether bone regeneration with xenograft is useful to prevent periodontal defects in the distal aspect of the M2Ms after the surgical extraction of the M3M.

## Material and Methods

This systematic review and meta-analysis was conducted in accordance with the statements of “Preferred Reporting Items for Systematic Reviews and Meta-Analyses” (PRISMA) (15).

- Eligibility criteria

The inclusion criteria were depicted in Table 1. We included articles that met the following eligibility criteria:

(P) Population: Non-smokers and healthy patients that underwent a M3M extraction.

**Table 1:**
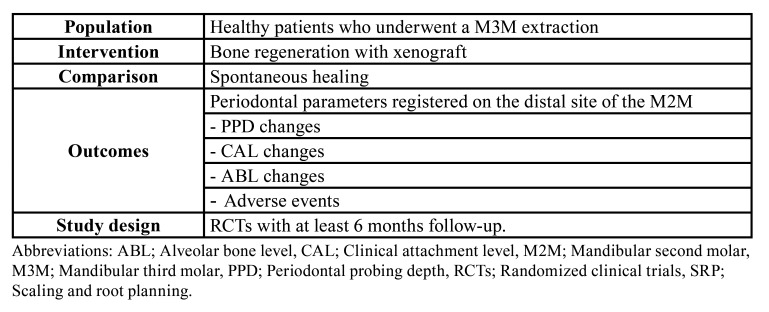
PICOS items. P; population, I; intervention, C; comparison, O; outcomes, S; study design.

(I) Intervention: Bone regeneration using bovine xenograft with or without membrane.

(C) Comparison: Spontaneous healing.

(O) Outcomes: Periodontal parameters evaluated on the distal site of the M2M. We registered the periodontal probing depth (PPD), the clinical attachment level (CAL), the alveolar bone level (ABL) the number of adverse events.

(S) Study design: Randomized clinical trials (RCTs) with at least 6 months follow-up. We did not apply any restriction in terms of language and year of publication.

According to these PICOS question, we designed the following clinical question: In non-smoker and healthy patients who need the removal of the M3M, what benefit does the use of bone regeneration with xenograft in terms of PPD, CAL, ABL and adverse events when compared to spontaneous healing have?

- Search strategy

Applying the following search strategy: (“molar, third” [MH] OR third molar* [TIAB] OR wisdom teeth* [TIAB] OR wisdom tooth* [TIAB] OR 3rd molar* [TIAB]) AND (“tooth extraction” [MH] OR extraction* [TIAB] OR removal* [TIAB] OR exodontia* [TIAB]) AND (“regeneration” [MH] OR “wound healing” [MH] OR “guided tissue regeneration, periodontal” [MH] OR “bone substitutes” [MH] OR guided bone regeneration* [TIAB] OR xenograft* [TIAB] OR “membranes, artificial” [MH] OR membrane* [TIAB] OR barrier* [TIAB]) NOT (“platelet rich fibrin”), Pubmed, Scopus and Cochrane Library databases were searched by two independent investigators (V.R-R. and J.T-S.) in April 2020. Additionally, we carried out a manual search of articles published during the last 10 years in “Medicina Oral Patología Oral y Cirugía Bucal”, “Journal of Oral and Maxillofacial Surgery”, “International Journal of Oral and Maxillofacial Surgery”, “Journal of Clinical Periodontology”, “Journal of Periodontology”, “Clinical Oral Investigations”, “Oral Surgery Oral Medicine Oral Pathology Oral Radiology”, “Journal of Dentistry" and "Journal of the American Dental Association”.

- Selection of studies

Two independent reviewers (V.R-R. and J.T-S.) carried out the selection of studies. After we removed the duplicates and the articles based on their title and abstracts, we selected the papers according to the inclusion criteria. Cohen’s kappa statistic was computed to measure the level of agreement between the two investigators.

Any disagreement during the article selection was resolved thanks to one independent investigator (MÁ.S-G.).

- Data extraction

The data extraction process was performed by two independent researchers (V.R-R. and J.T-S.). The extraction Tables included the name of the authors, country of origin, year of publication, study design, participant characteristics, surgical interventions, postoperative follow-up and the outcomes. Finally, we contacted with the authors of the selected studies for clarification when data were missing or incomplete.

- Risk of bias assessment

Two independent reviewers (V.R-R. and J.T-S.) evaluated the risk of bias of each article by means of "Cochrane Handbook for systematic reviews of interventions, version 5.1.0" (16). We evaluated as low, unclear or high risk of bias the following six quality criteria: random sequence generation, allocation concealment, patient blinding, outcome blinding, incomplete outcome data and selective reporting. Finally, a third independent reviewer (MÁ.S-G.) resolved any disagreement during this step.

- Statistical analysis

Odds ratio (OR) with 95% confidence intervals (CI) was used for adverse events outcome. In order to estimate the size of the effect, mean difference (MD) and standard deviation (SD) were used for PPD, CAL and ABL. A pairwise meta-analysis was conducted using RevMan software (Review Manager version 5.3; The Cochrane Collaboration, Copenhagen, Denmark) using M3M as the statistical unit in split-mouth studies. We selected the random effect model due to methodological and clinical heterogeneity expected across the included studies (17). In addition, significant heterogeneity was interpreted when I2 value was >50 (18). Statistical significance was defined as *P* < 0.05 for all analyses.

Forest plots were (JUSTIFICACIÓN DEL TEXTO) created to illustrate the effects in the meta-analysis of the global

estimation.

## Results

- Study selection and description

The initial electronic and manual search rendered 795 references. After the removal of the duplicates and the irrelevant articles based on their title and abstracts, 7 full texts were screened. Inter-reviewer agreement between the investigators (V.R-R. and J.T-S.) was 100% with a Cohen’s kappa index of 1 (perfect agreement).

**Figure 1:**
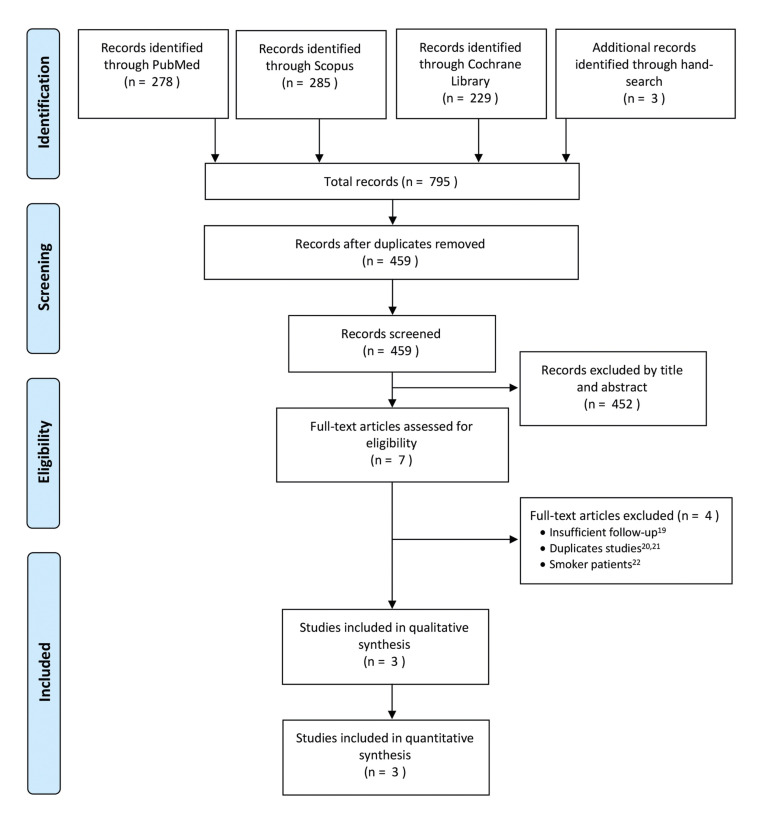
PRISMA flow chart of the study selection process.

The reasons for rejecting four articles were as follows: an insufficient follow-up (19), duplicates studies (20,21) and included smoker patients (22). Finally, for the present review three articles (23-25) were selected (Fig. 1).

- Risk of bias assessment

**Figure 2:**
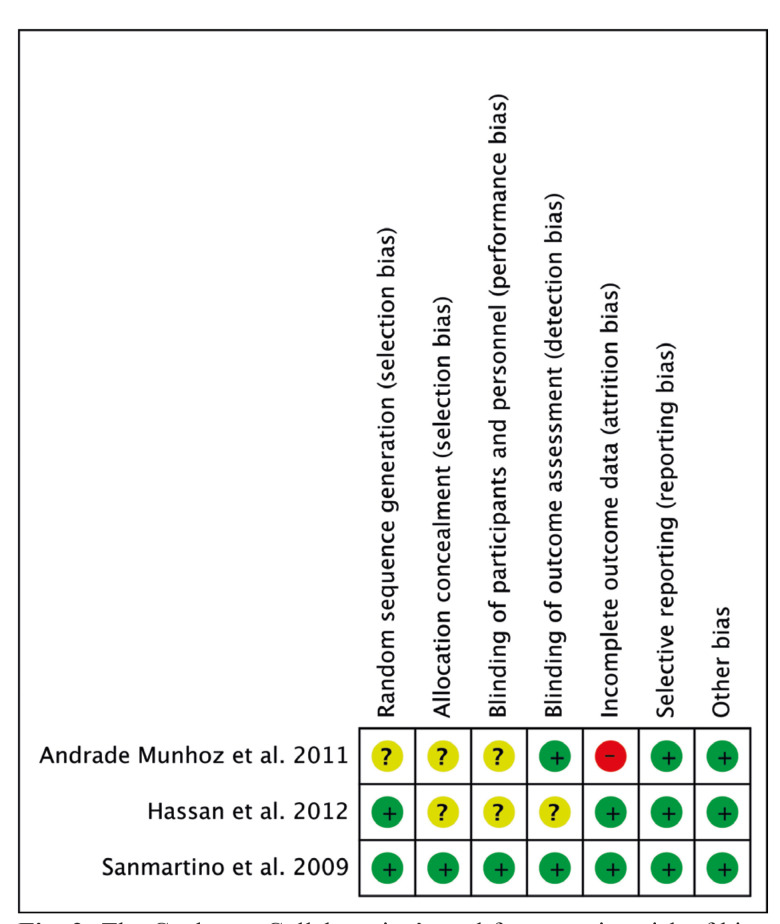
The Cochrane Collaboration’s tool for assessing risk of bias for randomized controlled trials.

As shown on Fig. 2, one article had a low risk of bias (25), while the studies published by Hassan *et al*. (24) and Andrade-Munhoz *et al*. (23) were classified as having unclear and high risk of bias, respectively.

- Extraction Data

We pooled the results of three articles (23-25) for assessing the xenograft alone or the xenograft covered by a collagen membrane after the removal of the M3M. The selected studies had a split-mouth design that comprised 98 patients (17 dropouts). Finally, this systematic review involves 81 patients with 162 M3Ms were included for the qualitative and quantitative analysis (Table 2).

**Table 2:**
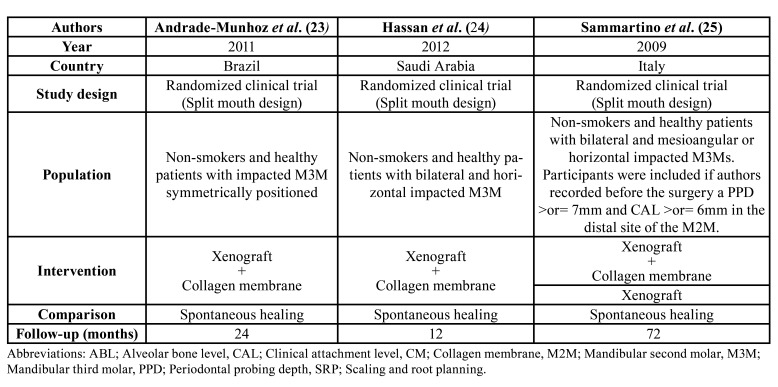
Description of the selected studies.

- Qualitative synthesis

Across the three included trials in the present review (23-25), one of these studies had a multi-arm design (25). All included papers compared xenograft plus collagen membrane versus spontaneous healing (23-25), while the multi-arm study had also a group comparing the xenograft without membrane (25).

In two trials (24,25), the xenograft covered or not by a collagen membrane revealed a statistically greater PPD and CAL gain than spontaneous healing at 12 months of follow-up after the M3M extraction (*P*<0.05).

Regarding ABL gain, one paper (23) reported higher values comparing xenograft covered by a collagen membrane (MD= 2.36; 95%CI 0.69 to 4.03; *P*=0.005) and spontaneous healing.

The results of the study by Sammartino *et al*. (25) showed that the xenograft plus the collagen membrane group had a significantly better results in terms of PPD and CAL than the group that used the xenograft alone (*P*<0.05).

None of the papers revealed statistically significant differences between groups with regard to adverse events. Out of the two studies that reported this outcome, three postoperative infections occurred in the grafted group and one in the control group (23).

- Quantitative synthesis

The same studies included in the qualitative synthesis were used to perform a pairwise meta-analysis comparing the use of xenograft covered by a collagen membrane after the removal of the M3M (23-25). We were unable to meta-analyse the adverse events outcome due to lack of data.

**Figure 3:**
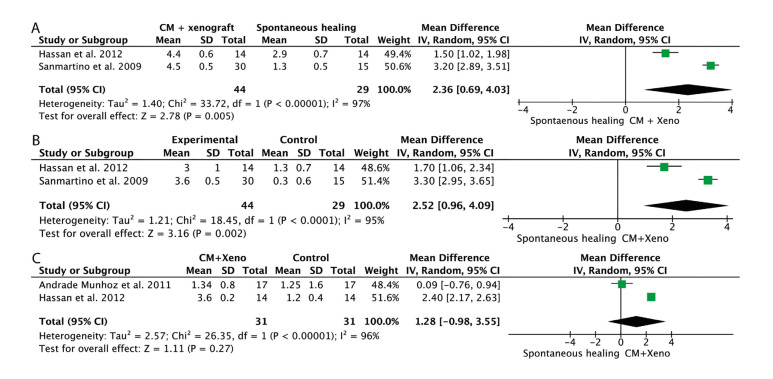
Forest plots for mean difference of periodontal probing depth reduction (PPD) (A), mean difference of clinical attachment level gain (CAL) (B) and mean difference of alveolar bone level gain (ABL) (C).

The results of two articles (24,25) were pooled for PPD and CAL analysis. These studies involved 73 M3Ms in total. Quantitative analysis favoured the use of the xenograft plus collagen membrane over the spontaneous healing in terms of PPD gain (MD= 2.36; 95%CI 0.69 to 4.03; *P*=0.005; I2=97%) (Fig. 3, Table 3) and CAL gain (MD=2.52; 95%CI 0.96 to 4.09; *P*=0.002; I2=95%) (Fig. 3, Table 3). No statistically significant differences were found in terms of ABL changes (Fig. 3, Table 3).

**Table 3:**
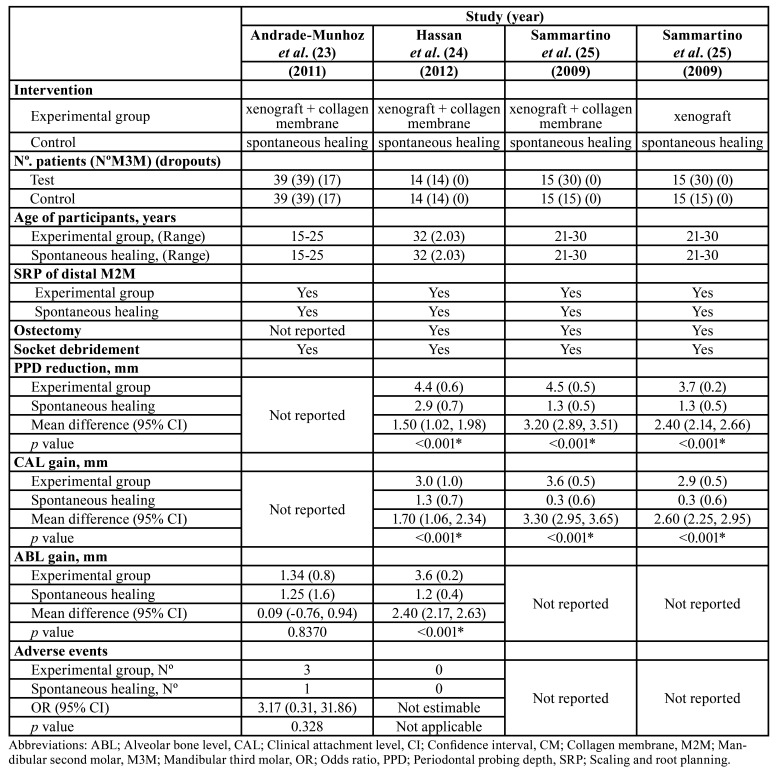
Comparison of the selected studies.

## Discussion

The purpose of the present study was to answer the following clinical question: in M3M post-extraction sites, what benefit does the use of bone regeneration with xenograft in terms of PPD, CAL, ABL and adverse events when compared to spontaneous healing have? After performing the meta-analysis, our results revealed a significant PPD reduction and CAL gain comparing guided bone regeneration (xenograft plus collagen membrane) versus spontaneous healing. Moreover, the bone filling with xenograft and the spontaneous healing resulted in similar ABL gain and number of postoperative complications.

Periodontal defect in the distal site of the M2M is a common finding in patients undergoing M3M extraction. In fact, through the selected studies, up to 50% of the cases exhibited PPD of at least 7mm before the intervention (24,25). This resembles the results of Garaas *et al*. (26) in which 65% of the patients had a PPD ≥4 mm at the distal site of the M2M.

The age of patients has their own relevance in bone regeneration of M3M sites. Kugelberg (27) demonstrated that patients older than 25 years old have a poor periodontal healing, which might cause periodontal pockets behind the M2M. In relation to this, the maximum age range of the patients included in our systematic review reaches 35 years old with impacted M3Ms, so they could obtain a greater benefit from xenograft bone regeneration to prevent future periodontal defects.

Over the years, different techniques (i.e. incision designs, soft-tissue suturing, scaling and root planning or periodontal regeneration) and materials (i.e. platelet concentrates, bone substitutes or occlusive membranes) have been investigated to solve this problem. A recent meta-analysis published by Chen *et al*. (28) showed a possible benefit of leaving a portion of gingiva around the M2M during the incision of the M3M extraction. Regarding other bone regeneration biomaterials, the results of this review have shown some discrepancies. Ge *et al*. (29) did not show significant results in PPD reduction and CAL gain at 6 and 12 months of follow-up with autologous bone substitute. Another autologous material that, unlike xenograft (23-25), has shown poor results in bone regeneration was platelet rich plasma (PRP), but instead, it has shown an improvement in soft tissue healing (30,31).

On the other hand, regarding the ABL gain, neither allograft (32,33) nor alloplastic (34) biomaterials showed significant improvements which could be in line with xenograft, specifically with the included study by Andrade-Munhoz *et al*. (23) In contrast, Hassan *et al*. (24) obtained a significant ABL gain with the use of xenograft at 12 months of follow-up. It should be noted that Andrade-Munhoz *et al*. (23) used a new type of xenograft that is only marketed in Brazil, unlike the other 2 articles analysed in this systematic review (24,25), which used a type of well-known xenograft, supported by numerous studies.

Regarding the use of resorbable or non-absorbable membrane there are no statistically significant differences between them, however, second surgery is avoided when the resorbable membranes are used (35,36). In this review, only one study (25) compared the xenograft with or without membrane and the best outcomes were for the membrane group.

Generally, bone regeneration increases the risk of postoperative complications (29,32,34), however, among the included studies, we did not obtain significant complications (23).

Although it would be interesting to obtain histological studies to observe whether tissue regeneration is formed, it is not clinically relevant since the objective of bone regeneration is that the patients do not have periodontal defects, being able to maintain sTable over time. Across the included studies, only one of them (25) provided histological results showing that with the use of a collagen membrane the level of xenogeneic particles was lower and more mature osteoid matrix (better bone quality) was observed at 6 months. Nevertheless, it is not essential since we have not evaluated this outcome.

In this review, all included studies (23-25) performed a scaling and root planning either in experimental or control groups. This procedure has been shown to remove plaque and calculus behind M2M and it consequently improves periodontal healing (37) therefore, the included studies could have been benefited from this procedure.

There were several limitations related to the present study that must be mentioned. Firstly, only three papers which compared the guided bone regeneration with xenograft and the spontaneous healing were able to be included in our meta-analysis. There were no studies to compare by a meta-analysis the effectiveness of the xenograft without a collagen membrane. Moreover, the limited number of patients and M3M included together with the fact that only one paper of the selected studies had a low risk of bias, did not allow to make robust conclusions. Another possible drawback of this meta-analysis was the substantial heterogeneity across the selected studies. Thus, authors recommend being cautious with the results of the present review.

## Conclusions

Within the above-mentioned limitations, it can be concluded that guided bone regeneration with xenograft and collagen membrane exhibited greater PPD reduction and CAL gain in the distal aspect of the M2M after the surgical extraction of the M3M than spontaneous healing. However, to confirm our results well-conducted investigations with larger samples and with a longer follow-up are needed.
